# Evaluation of reference genes for quantitative expression analysis in *Mylabris sibirica* (Coleoptera, Meloidae)

**DOI:** 10.3389/fphys.2024.1345836

**Published:** 2024-04-08

**Authors:** Chen-Hui Shen, Min Tang, Xiao-Fei Li, Li Zhu, Wei Li, Pan Deng, Qing Zhai, Gang Wu, Xiao-Hong Yan

**Affiliations:** ^1^ Key Laboratory of Agricultural Genetically Modified Organisms Traceability, Ministry of Agriculture and Rural Affairs, Oil Crops Research Institute of Chinese Academy of Agricultural Science/Supervision and Test Center (Wuhan) for Plant Ecological Environment Safety, Ministry of Agriculture and Rural Affairs, Wuhan, China; ^2^ Northern Propagation Experiment Station, Center for Science and Technology Dissemination and Industrial Development, Oil Crops Research Institute of Chinese Academy of Agricultural Science, Wuhan, China; ^3^ Institute of Leisure Agriculture, Jiangsu Academy of Agricultural Sciences, Nanjing, China; ^4^ College of Plant Protection, Henan Agricultural University, Zhengzhou, China

**Keywords:** *Mylabris sibirica*, reference gene, RT-qPCR, RefFinder, geNorm

## Abstract

*Mylabris sibirica* is a hypermetamorphic insect whose adults feed on oilseed rape. However, due to a shortage of effective and appropriate endogenous references, studies on molecular functional genes in *Mylabris sibirica*, have been tremendously limited. In this study, ten internal reference genes (*ACT*, *ARF1*, *AK*, *EF1α*, *GAPDH*, *α-TUB*, *RPL6*, *RPL13*, *RPS3* and *RPS18*) were tested and assessed under four selected treatments including adult ages, adult tissues, temperatures, and sex by RT-qPCR based on five methods (Ct value, geNorm, NormFinder, BestKeeper and RefFinder). Our findings showed that *RPL6* and *RPL13* were the most optimal internal reference gene combination for gene expression during various adult ages and under diverse temperatures; The combination of *RPL6* and *RPS18* was recommended to test gene transcription levels under different adult tissues. *AK* and *RPL6* were the best reference genes in male and female adults. *RPL6* and *RPL13* were the most appropriate reference gene pair to estimate gene expression levels under four different tested backgrounds. The relative transcript levels of a uridine diphosphate (UDP)-N-acetylglucosamine-pyrophosphorylase (*MsUAP*), varied greatly according to normalization with the two most- and least-suited reference genes. This study will lay the basis for further molecular physiology and biochemistry studies in *M. sibirica*, such as development, reproduction, sex differentiation, cold and heat resistance.

## Introduction

Quantitative real-time fluorescent polymerase chain reaction (qRT-PCR) is a crucial method to measure target gene transcripts (Derveaux et al., 2010; Jozefczuk and Adjaye, 2011) and microbial abundance (Ali et al., 2018a; Ali et al., 2018b; Ali et al., 2018c; Ali et al., 2019) due to its high specificity, sensitivity and accuracy. Its precision is affected by numerous biological and technical factors, such as RNA purity, PCR efficiency, inappropriate data and statistical analyses (Valasek and Repa, 2005; Bustin et al., 2009). When qRT-PCR is applied to test transcripts, it is indispensable to normalize to improve the quantitative results by combining relatively stable reference genes (Bustin et al., 2009). If poor internal genes are used, the quantitates of nucleic acid will be inaccurate (Yang et al., 2014). Hence, Suitable internal references should be verified under diverse backgrounds, including developing stages, tissues and hosts (Andersen et al., 2004; Zhang et al., 2022; Shen et al., 2022).

As usual, reference genes are housekeeping genes (HKGs), whose expression levels are stable during different physiological states or in different cells (Zhang et al., 2022). At present, the 10 most generally used references contain actin (*ACT*), glyceraldehyde-3-phosphate dehydrogenase (*GAPDH*), ribosomal protein (*RPL* and *RPS*), TATA binding protein (*TATA*), heat shock protein (*HSP*), elongation factor 1α (*EF1α*), tubulin (*TUB*), 18S ribosomal RNA (*18S*) and succinate dehydrogenase complex subunit A (*SDHA*) (Lü et al., 2018). In addition, several novel methods including bioinformatic transcripome analysis have been used for reference gene screening (Yu et al., 2020). In previously researches, one to five analysis tools were applied to evaluate gene expression stability (Lü et al., 2018). Currently, the RefFinder is the only web-based tool available for evaluating candidate housekeeping genes by integrating the four available computational programs (geNorm, NormFinder, BestKeeper, and ΔCt method) into a web-based tool (Xie et al., 2023).

Nevertheless, there is no one stable internal gene under numerous tested treatments, including developmental stages, tissues and hosts (Andersen et al., 2004; Zhang et al., 2022; Shen et al., 2022; Shen et al., 2023). To evaluate accurate gene expression levels, each candidate reference gene under diverse tested conditions must be validated (Xu et al., 2021).

*Mylabris sibirica* (Coleoptera, Meloidae) is a hypermetamorphic pest that leads to significant losses in oilseed rape (*Brassica napus*) production (Tian Yubo et al., 2021). Its adults mainly feed on flowers of *B. napus* (Tian Yubo et al., 2021). Until now, studies on *M. sibirica* have concentrated on classification (ČernÝ and Vrabec, 2019; Pan and Ren, 2020), phylogenetics (Bologna et al., 2005) and medical value (Tian Yubo et al., 2021), however, little is known on gene functions. Gene expression analyses are essential to study molecular mechanisms of development, reproduction and physiology. Nevertheless, no study evaluating *M. sibirica* gene expression analysis has been reported. In order to further manage *M. sibirica* based on novel target genes, screening for optimal reference genes is imperative.

Nowadays, many researches have reported the optimal reference genes in Coleopterans (Shi et al., 2013; Pan et al., 2015; Sang et al., 2015; Lü et al., 2018; Yang et al., 2018; Ma et al., 2021; Sellamuthu et al., 2021; Zhang et al., 2022; Sellamuthu et al., 2022). For example, the most optimal internal references have been published in *Phyllotreta striolata* (*EF1A* and *VATPA*) (Guo et al., 2023), *Henosepilachna vigintioctomaculata* (*RPS18* and *RPL13*) (Zhang et al., 2022) and *Phaedon brassicae* (*RPL32* and *EF1α* in various tissues, *RPL19* and *TBP* across diverse developmental stages, *RPL32* and *RPL19* under insecticide exposure*, RPL32* and *TBP* under thermal stress) (Ma et al., 2021). In general, at least two reference genes are used to validate expression levels in each insect species (Zhang et al., 2022).

Since top 10 most frequently used reference genes contains *ACT*, *RPL*, *TUB*, *GAPDH*, *RPS*, *18S*, *EF1*α, *TATA*, *HSP* and *SDHA* in insects and they are the most optimal internal references in adverse experimental backgrounds (Lü et al., 2018). In this study, ten candidate references, i.e., *ACT*, *ARF1*, *AK* (arginine kinase), *EF1α*, *GAPDH*, *α-TUB*, *RPL6*, *RPL13*, *RPS3* and *RPS18* were accordingly selected to assess the stability of gene expression during various adult ages, among various adult tissues, under diverse temperatures, and in females and males in *M. sibirica*. Finally, uridine diphosphate (UDP)-N-acetylglucosamine-pyrophosphorylase (*UAP*) was used as the target gene to verify our findings. Our results will offer the reference foundation for further molecular mechanisms in *M. sibirica*.

## Methods and materials

### Insect collection

*M. sibirica* adults used in this research were collected from *B. napus* plants at the Northern Propagation Experiment Station of the Oil Crops Research Institute, Chinese Academy of Agricultural Sciences, located in Haidong city, Qinghai Province, China in 2023 (coordinates: 36°31′10.47″N, 101°59′7.40″E). The adults were fed in an insectary for 1 week at 25°C ± 2 °C, 16 h:8 h photoperiod and 55% ± 5% relative humidity using oilseed rape as food.

### Specimens through diverse adult ages

The newly emerged adults were grouped as males and females and kept in different net cages (30 cm × 30 cm × 40 cm, hand-made). For each biological replicate, a total of five adults (three males and two females) were collected every 24 h for a continuous period of 72 h. Three biological replicates were conducted.

### Samples in different adult tissues

Epidermis, foregut, midgut, hindgut, trachea and antenna were dissected from the newly-emerged adults. Ten adults (5 males and 5 females) were dissected for one biological replicate. Three biological replicates were prepared.

### Specimens under various temperatures

The newly-emerged adults were reared under five temperatures (4 °C, 15 °C, 20 °C, 26 °C and 35 °C) for 8 h. Five adults (3 males and 2 females) were collected for one biological replicate. Three biological replicates were prepared.

### Collections in males and females

The newly-emerged male and female adults were kept in an insectary at 26 °C for 5 days. Five males and females were collected respectively for one biological replicate. Three biological replicates were prepared.

### Total RNA extraction and cDNA synthesis

Total RNAs were extracted by TRIzol reagent (YiFeiXue Tech, Nanjing, China) following the manufacturer’s instructions. The RNA integrity was assessed by electrophoresis on a 1.5% agarose gel. The purity and concentration of the total RNA samples were determined using a NanoDrop ND-1000 spectrophotometer (Nanodrop Technologies, Rockland, DE, United States), with 260/280 and 260/230 ratios ranging from 1.9 to 2.1. The HiScript III RT SuperMix (containing gDNA wiper, Vazyme Biotech Co.,Ltd., Nanjing, China)was used to synthesize cDNA. The reaction mixtures were incubated at 37°C for 15 min, followed by 85°C for 5 s. The resulting cDNA samples were diluted 5-fold for the pursuant PCR and RT-qPCR.

### Selection and identification of candidate references

Ten HKG sequences (*actin*, *ACT*; *α-tubulin*, *α-TUB*;A*DP ribosylation factor 1*, *ARF1*; *elongation factor 1α*, *EF1α*; *arginine kinase*, *AK*; *ribosomal proteins RPL6*, *RPL13*, *RPS3* and *RPS18*; *glyceraldehyde-3-phosphate dehydrogenase*, *GAPDH*) were selected by BioEdit software based on the transcriptome data of *M. sibirica* (unpublished data). The resulting HKG information was presented in Supplementary Table S1.

The primers of Reverse transcriptase PCR (RT-PCR) were designed by Primer Premier 5.0 and performed according to the previously described method (Shen et al., 2022). The resultant sequences were submitted to GenBank. The accession numbers were located in Supplementary Table S1.

### Quantitative real-time PCR (qRT-PCR)

The primers of qRT-PCR were designed by NCBI Primer-BLAST, and were listed in Table 1. The qRT-PCR reactions were conducted using ChamQ Universal SYBR qPCR Master Mix (Vazyme Biotech Co., Ltd.) and CFX96 Real-Time System (Bio-Rad Laboratories, Hercules, CA, United States). The reaction mixture consisted of a 20 μL reaction volume including 1 μL cDNA template, 0.4 μL of forward primer (10 μM), 0.4 μL of reverse primer (10 μM), 10 μL of 2× ChamQ Universal SYBR qPCR Master Mix and 8.2 μL of RNase Free water. The qRT-PCR reaction conditions consisted of the following steps: an initial step of 95°C for 30 s, followed by 40 cycles of denaturation at 95°C for 5 s, and subsequent annealing at 60°C for 34 s, followed by one cycle of 95 °C for 15 s, 60 °C for 60 s and 95 °C for 1 s. The specificity of PCR amplicons were assessed by a melting curve analysis, analyzing by the BioRadCFXManager and gel electrophoresis. All experiments were repeated in triplicate. Amplification efficiencies (E) were calculated using a 5-fold dilution series of template via the following equation: E = (10^[−1/slope]^ − 1) × 100%.

**TABLE 1:** Primers of 10 candidate house-keeping genes used in qRT-PCR.

Gene	Primer sequences (5′to 3′)	Length (bp)	Slope	R2	Efficiency (%)
*ACT*	F-GTGTGACGAAGAAGTTGCTGC	101	−3.352	0.995	98.76
	R-TTGATGGGAAAACAGCACGC				
*ARF1*	F-ACATCAGGCGTTAGGTTTGG	81	−3.560	0.998	90.94
	R-AAACCTTCACCCTTCGTTGC				
*AK*	F-TGGTTGACGCTGCTGTTTTG	217	−3.558	0.996	91.01
	R-GCTTCTGCATCAGGAGCGTA				
*α-TUB*	F-CAGTCCATGTCGGTCAAGCC	88	−3.424	0.999	95.91
	R-TGTCCATCAGGTTGGATGCC				
*GAPDH*	F-CGGTTTTGGCCGTATTGGTC	146	−3.340	0.992	99.25
	R-CACCTTTAAAATGGCCGTGTGT				
*EF1α*	F-AAGAAGGCAAAGCTGACGGT	239	−3.402	0.999	96.76
	R-GGCTTCGTGGTGCATTTCAA				
*RPL6*	F-AGAAAACGAAAGCAACACCGAA	239	−3.465	1	94.36
	R-GCCTTTATGAGCGCCTGCTA				
*RPL13*	F-TGCTATTGCCCCAAGACCTG	91	−3.541	0.994	91.61
	R-ACGTCCAGCCCTAACTTTCG				
*RPS3*	F-ATACAGCAACACGCCACGTA	163	−3.547	0.998	91.39
	R-GCTGGACCTAGTGGAACGAC				
*RPS18*	F-GTCAGCTCACATCGGCTACA	80	−3.465	1	94.36
	R-ACCTCTATGGGCACGGATCT				

Note: ACT, actin; ARF1, ADP, ribosylation factor 1; AK, arginine kinase; α-TUB, α-tubulin; GAPDH, glyceraldehyde-3-phosphate dehydrogenase; EF1α, elongation factor 1α; RPL6, RPL13, RPS3 and RPS18, ribosomal protein.

### Stability determination of candidate reference genes

Uridine diphosphate (UDP)-N-acetylglucosamine-pyrophosphorylase (UAP) of *M. sibirica* was used to verify the stability of candidate reference genes (GenBank: OR838722). The primer sequence of the target gene was as follows:

Forward: ATT​ATT​GAT​GGC​CGG​TGG​TC

Reverse: ACC​ATT​TAA​ACC​GGT​CTT​TTG​TT

Based on the stability (*RPL6* and *RPS18*) and instability (*AK* and *GAPDH*) of primary reference genes, the relative levels of *MsUAP* in different adult tissues were computed by the 2^−ΔΔCT^ method and from three replicates. One-way analysis of variance was used to assess significance in *UAP* expression levels among various adult tissues (SPSS, Chicago, IL, United States).

### Data processing

The raw Ct values were obtained using the BioRadCFXManager. The stability of candidate HKGs were measured using the ΔCt method (Silver et al., 2006), geNorm (Vandesompele et al., 2002), Normfinder (Andersen et al., 2004) and BestKeeper (Pfaffl et al., 2004). Furthermore, the optimal number of reference genes for gene expression normalization was determined by pairwise variation (V_n/n+1_) using the GeNorm program Typically, a V_n/n+1_ value below the threshold of 0.15 indicates that the starting n reference genes are enough for normalizing target gene expression. Lastly, the overall ranking of each experimental background was assessed based on RefFinder (Xie et al., 2012; Xie et al., 2023).

## Results

### Selection of candidate reference genes

Ten HKG genes, i.e., *ACT*, *ARF1*, *AK*, *EF1α*, *GAPDH*, *α-TUB*, *RPL6*, *RPL13*, *RPS3* and *RPS18* in *M. sibirica* were selected. The obtained sequences were submitted to GenBank; the accession numbers were shown in Supplementary Table S1.

The products obtained from qRT-PCR were validated through sequencing. The primer specificity for qRT-PCR was confirmed through melting curve analysis. As expected, slopes of all primer pairs were less than −3.0, and regression coefficient (R2) and efficacy values ranged from 0.992-1 and 90.94%–99.25%, respectively (Table 1). Our findings demonstrated that efficiency met the required standards for traditional qRT-PCR (Bustin et al., 2009).

### Stability of the ten HKGs

Samples were collected from three adult ages (the first to third days of the newly-emerged adults), six adult tissues (foregut, midgut, hindgut, epidermis, trachea and tentacle), five temperature treatments (4 °C, 15 °C, 20 °C, 26 °C and 35 °C) and both sexes (5 days after emerging). We discovered that the mRNA levels of ten HKGs were abundant at various adult ages, during diverse adult tissues, under different temperatures and both sexes using qRT-PCR,.

**FIGURE 1:**
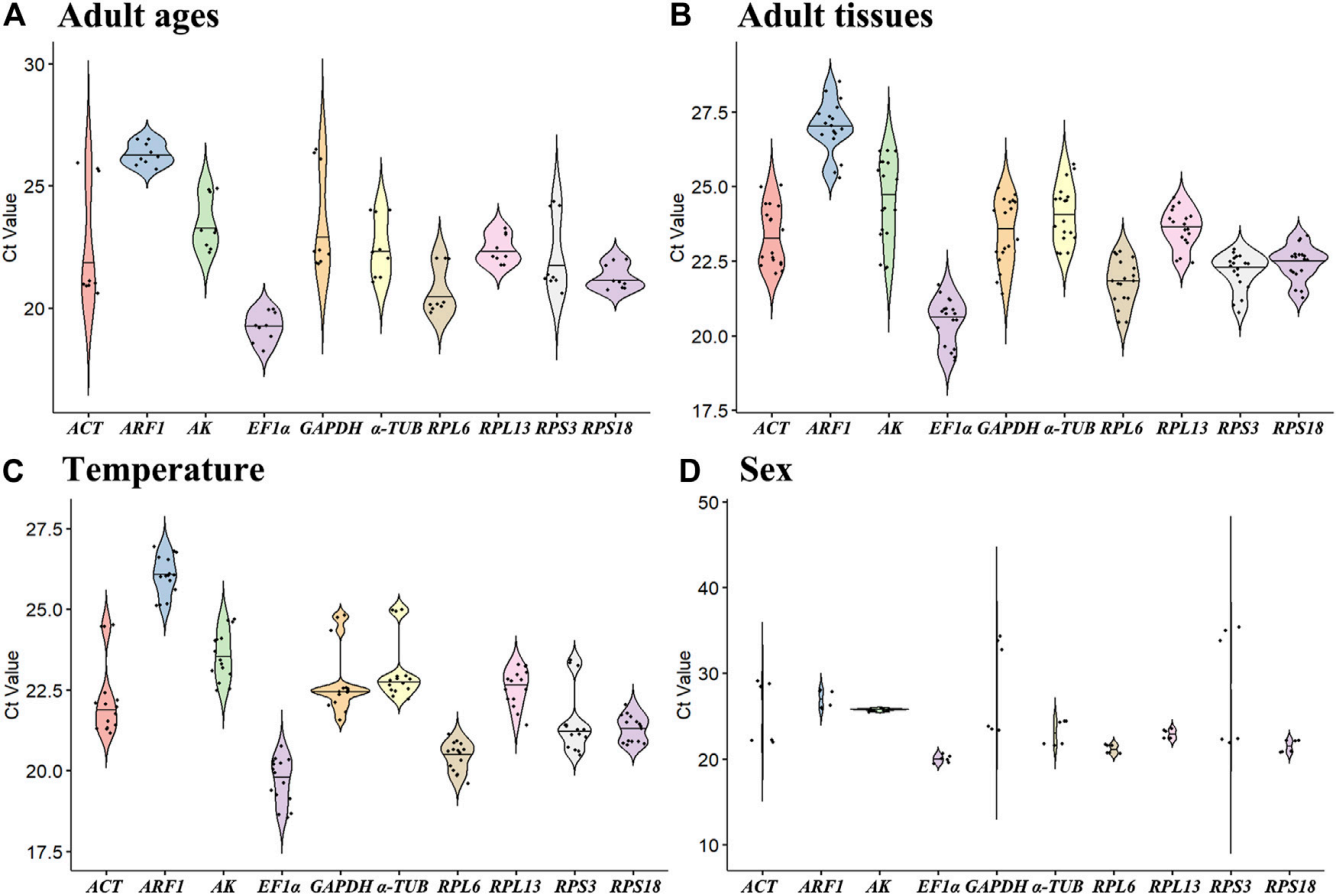
Expression levels of ten house-keeping genes in *Mylabris sibirica*. The mean C_t_ values for 10 candidate reference genes are shown in four different experiments: **(A)** adult ages, **(B)** adult tissues, **(C)** temperature, **(D)** sex. Mean Ct values for the eight candidate reference genes are presented in box plots, where each box indicates the 25th and 75th percentiles, and the line across the box represents the median. Abbreviation: ACT, actin; ARF1, ADP ribosylation factor 1; AK, arginine kinase; α-TUB, α-tubulin; GAPDH, glyceraldehyde-3-phosphate dehydrogenase; EF1α, elongation factor 1α; RPL6, RPL13, RPS3 and RPS18, ribosomal protein. The abbreviations are exactly the same as Figure 2 through Figure 6.

The all cycle threshold values (Ct) under diverse tested backgrounds were presented (Figure 1). The ΔCt method assesses the genes stability based on genes average of STDEV (Silver et al., 2006). During diverse adult ages, the expression fluctuations of *RPL6* and *AK* were smaller, whereas *ACT* and *GAPDH* were higher (Figure 1A). Under diverse tissues, except for *AK*, the expression variations were small in ten HKGs (Figure 1B). Under various temperatures, the expression difference of *RPL13* and *ARF1* was smaller (Figure 1C). In males and females, the expression variations of *AK* and *EF1α* were small in ten HKGs (Figure 1D).

### Stability of the ten HKGs under adult ages

**FIGURE 2:**
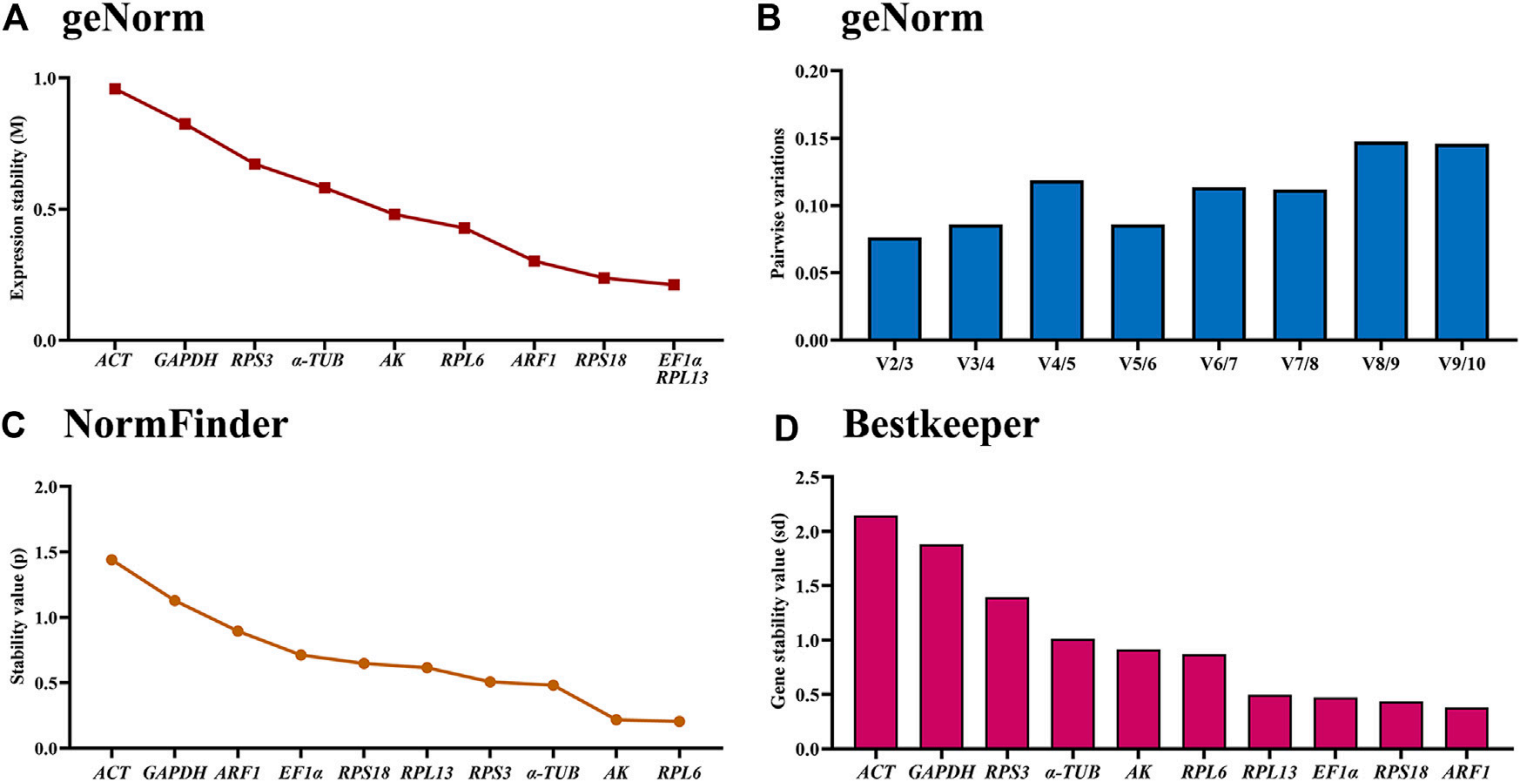
Stability of the ten house-keeping genes in *Mylabris sibirica* during diverse adult ages. All stages of *Mylabris sibirica* adults were sampled (collected on the first to third days of the newly-emerged adults). The expression stability rankings are determined by geNorm, NormFinder and BestKeeper.

The geNorm statistical analysis assesses the gene stability by M-values (the average expression stability) and V-values (pairwise variation). These results showed that *EF1α* and *RPL13* were the most stable internal genes during various adult ages, with M-values below 0.25. In contrast, the most unstable genes were *ACT* and *GAPDH* (Figure 2A; Table 2). Pairwise variation analysis showed that all values were below 0.15, demonstrating that two reference genes were needed for the gene expression analysis under adult ages (Figure 2B).

**TABLE 2:** Expression stability of the candidate reference genes under different experimental conditions.

Conditions	CRGs^a^	geNorm		Normfinder		BestKeeper		ΔCt	
		Stability	Rank	Stability	Rank	Stability	Rank	Stability	Rank
Developmental stages	*ACT*	0.959	9	1.441	10	2.146	10	1.496	10
	*ARF1*	0.302	3	0.895	8	0.378	1	0.998	8
	*AK*	0.480	5	0.217	2	0.913	6	0.782	2
	*α-TUB*	0.581	6	0.482	3	1.013	7	0.892	6
	*GAPDH*	0.825	8	1.129	9	1.884	9	1.257	9
	*EF1α*	0.211	1	0.712	7	0.472	3	0.876	5
	*RPL6*	0.428	4	0.205	1	0.873	5	0.726	1
	*RPL13*	0.211	1	0.615	5	0.497	4	0.822	3
	*RPS3*	0.672	7	0.507	4	1.395	8	0.907	7
	*RPS18*	0.237	2	0.648	6	0.435	2	0.833	4
Adult tissues	*ACT*	0.494	6	0.434	5	0.923	8	0.729	6
	*ARF1*	0.396	5	0.583	8	0.632	5	0.750	7
	*AK*	0.769	9	1.295	10	1.236	10	1.354	10
	*α-TUB*	0.556	7	0.525	7	0.841	7	0.783	8
	*GAPDH*	0.623	8	0.614	9	0.989	9	0.852	9
	*EF1α*	0.345	4	0.404	4	0.632	6	0.665	4
	*RPL6*	0.298	3	0.304	1	0.598	4	0.604	1
	*RPL13*	0.253	2	0.401	3	0.522	3	0.635	2
	*RPS3*	0.235	1	0.493	6	0.491	2	0.684	5
	*RPS18*	0.235	1	0.388	2	0.470	1	0.639	3
Temparature treatment	*ACT*	0.632	9	0.700	10	0.907	10	0.784	9
	*ARF1*	0.259	2	0.368	2	0.457	3	0.565	2
	*AK*	0.593	8	0.624	9	0.645	6	0.775	8
	*α-TUB*	0.514	6	0.420	5	0.745	8	0.600	6
	*GAPDH*	0.497	5	0.424	6	0.753	9	0.598	5
	*EF1α*	0.283	3	0.429	7	0.615	5	0.598	5
	*RPL6*	0.233	1	0.391	4	0.378	2	0.567	3
	*RPL13*	0.233	1	0.311	1	0.469	4	0.541	1
	*RPS3*	0.438	4	0.383	3	0.737	7	0.581	4
	*RPS18*	0.540	7	0.618	8	0.352	1	0.708	7
Sex	*ACT*	1.661	7	2.246	7	3.328	8	3.593	8
	*ARF1*	0.371	4	2.584	8	0.947	6	2.779	7
	*AK*	0.501	5	1.104	2	0.109	1	2.324	1
	*α-TUB*	0.922	6	0.678	1	1.335	7	2.618	6
	*GAPDH*	2.468	8	4.474	9	5.037	9	4.881	9
	*EF1α*	0.256	3	1.789	3	0.345	2	2.369	2
	*RPL6*	0.160	1	1.940	4	0.469	3	2.377	3
	*RPL13*	0.160	1	1.961	5	0.484	4	2.391	4
	*RPS3*	3.187	9	5.991	10	6.260	10	6.063	10
	*RPS18*	0.187	2	2.158	6	0.631	5	2.477	5

^a^ Candidate reference gene.

Based on the NormFinder algorithm, the stable rankings of ten internal genes from the most stable to the least were *RPL6*, *AK*, *α-TUB*, *RPS3*, *RPL13*, *RPS18*, *EF1α*, *ARF1*, *RPL27*, *GAPDH* and *ACT* (Figure 2C; Table 2). Except for *GAPDH* and *ACT*, the *p* values of other genes were had less than 1 (Figure 2C; Table 2).

According to the BestKeeper analysis, *ARF1*, *RPS18*, *EF1α*, *RPL13*, *RPL6*, *AK* were the most stable, whose standard deviation (SD) values from raw Ct values were 0.378, 0.435, 0.472, 0.497, 0.873 and 0.913, respectively. In constrast, the SD values of *α-TUB*, *RPS3*, *GAPDH* and *ACT* were more than 1.0 (Figure 2D; Table 2).

The stability of the ten HKGs were compared and ordered by an online tool RefFinder: *RPL6*>*RPL13*>*RPS18*>AK>*ARF1>EF1α*>*α-TUB*>*RPS3*>*GAPDH*>*ACT* (Figure 6A). Thus, *RPL6* and *RPL13* are the optimal internal gene pair to calculate gene expression during various adult ages (Table 3).

**TABLE 3:** A list of the recommended reference genes in *M. sibirica* for different experimental conditions.

Experimental conditions	The recommended reference genes	
Development stages	*RPL6*	*RPL13*
Adult tissues	*RPL6*	*RPS18*
Temperature	*RPL6*	*RPL13*
Sex	*RPL6*	*AK*
All samples	*RPL6*	*RPL13*

Note: AK, arginine kinase; RPL6, RPL13, RPS3 and RPS18, ribosomal protein.

### Stability of the ten HKGs during different adult tissues

**FIGURE 3:**
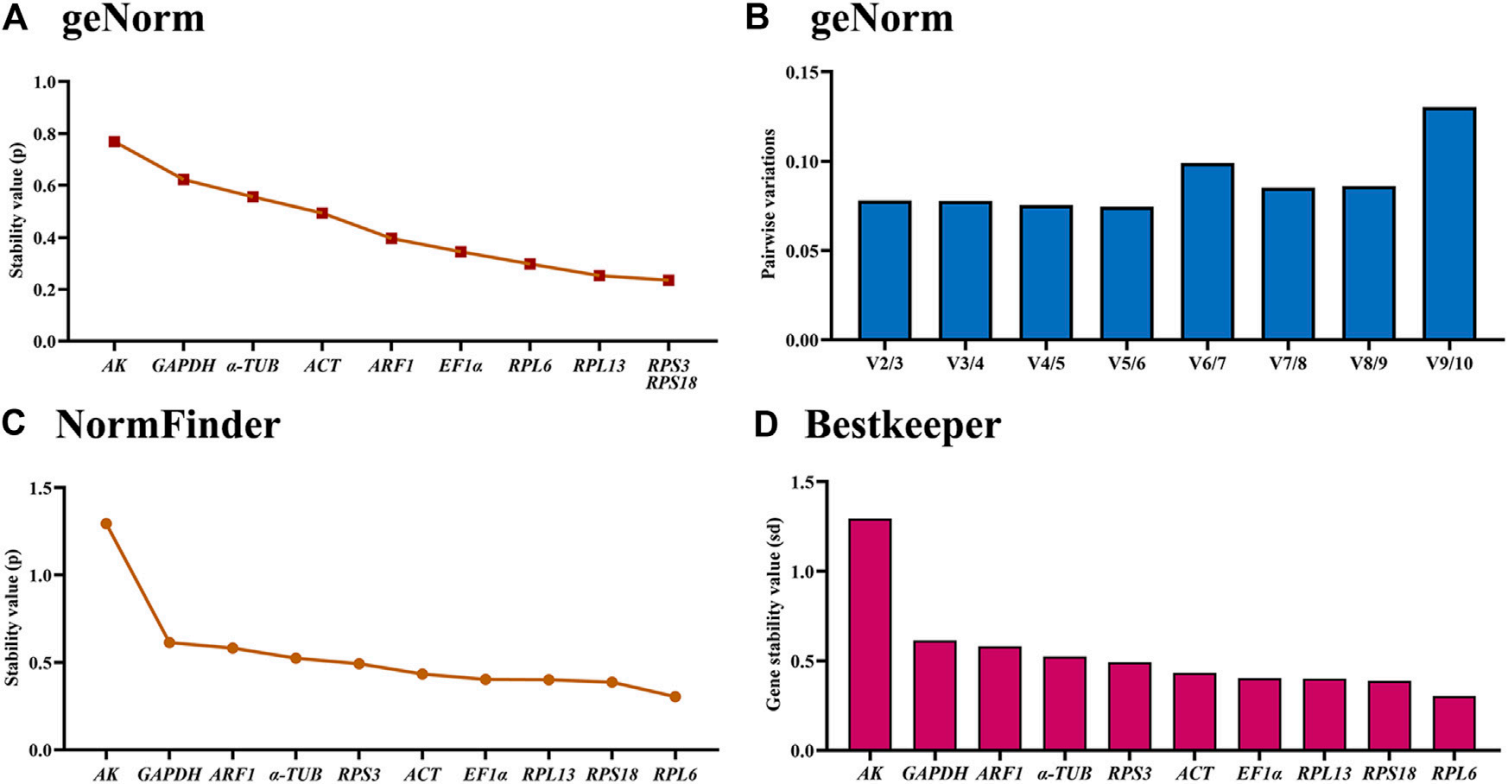
Stabilities of the ten house-keeping genes in *Mylabris sibirica* among various adult tissues. Foregut, midgut, hindgut, epidermis, trachea and antenna were dissected from the newly-emerged adults. The expression stability rankings are determined by geNorm, NormFinder and BestKeeper.

According to the geNorm data, the reference genes rankings ranging from the most stable to the least stable were *RPS3*, *RPS18*, *RPL13*, *RPL6*, *EF1α*, *ARF1*, *ACT*, *α-TUB*, *GAPDH* and *AK* (Figure 3A; Table 2). Pairwise variation analysis showed that all values were less than 0.15, indicating that two reference genes from different tissues were needed for the gene expression analysis (Figure 3B).

Based on the NormFinder result, the reference genes rankings were as follows: *RPL6*>*RPS18*>*RPL13*>*EF1α*>*ACT*>*RPS3*>*α-TUB*>*ARF1*>*GAPDH*>*AK* (Figure 3C; Table 2). Again, except for *AK*, the *p* values of other reference genes were below 1.0 (Figure 3C; Table 2).

The BestKeeper analysis showed that *RPL6*, were the most stable, with the SD values of 0.604 (Figure 3D; Table 2). Again, except for *AK*, the SD values of other reference genes were below 1.0 (Figure 3D; Table 2).

RefFinder provided a thorough ranking order ranging from the most stable to the least stable:*RPS18*>*RPL6*>*RPL13*>*RPS3*>*EF1α*>*ARF1*>*ACT*>*α-TUB*>*GAPDH*>*AK* (Figure 6B). Thus, *RPL6* and *RPS18* are the most suitable internal gene pair to measure gene expression under different adult tissues (Table 3).

### Expression stability of the ten HKGs under various temperature treaments

**FIGURE 4:**
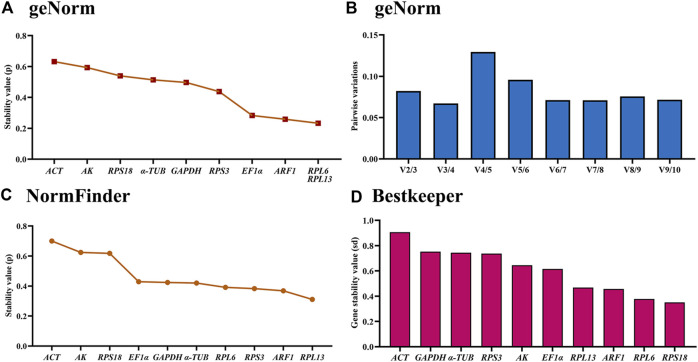
Stability of the ten house-keeping genes in *Mylabris sibirica* under different temperatures. The newly-emerged adults reared under five temperatures (4°C, 15°C, 20°C, 26 °C and 35 °C) were collected. The expression stability rankings are determined by geNorm, NormFinder and BestKeeper.

The geNorm analysis showed that *RPL6*, *RPL13*, *ARF1* and *EF1α* were the most stable internal genes under different temperatures, whose M-values were 0.233, 0.233, 0.259 and 0.283, respectively (Figure 4A; Table 2). Moreover, pairwise variation analysis showed that all values were less than 0.15, indicating that two different internal genes are needed for testing gene expression during various temperature treaments (Figure 4B).

The NormFinder data uncovered that the steady rankings were *RPL13*, *ARF1*, *RPS3*, *RPL6*, *α-TUB*, *GAPDH*, *EF1α*, *RPS18*, *AK* and *ACT* (Figure 4C; Table 2).

BestKeeper data showed that *RPS18*, *RPL6*, *ARF1* and *RPL13* were the most optimal genes, whose SD values were 0.352, 0.378, 0.457 and 0.469, respectively (Figure 4D; Table 2). Again, these genes showed values less than 0.5, suggesting their stabilities were similar.

Based on the RefFinder data, the stability orders were as follows: *RPL13*>*RPL6*>*ARF1*>*RPS3*>*RPS18*>*EF1α*>*GAPDH*>*α-TUB* >*AK*>*ACT* (Figure 6C). Thus, *RPL6* and *RPL13* are the best internal gene pair for assessing mRNA levels of genes under various temperature treaments (Table 3).

### Stability of the ten HKGs in males and females

**FIGURE 5:**
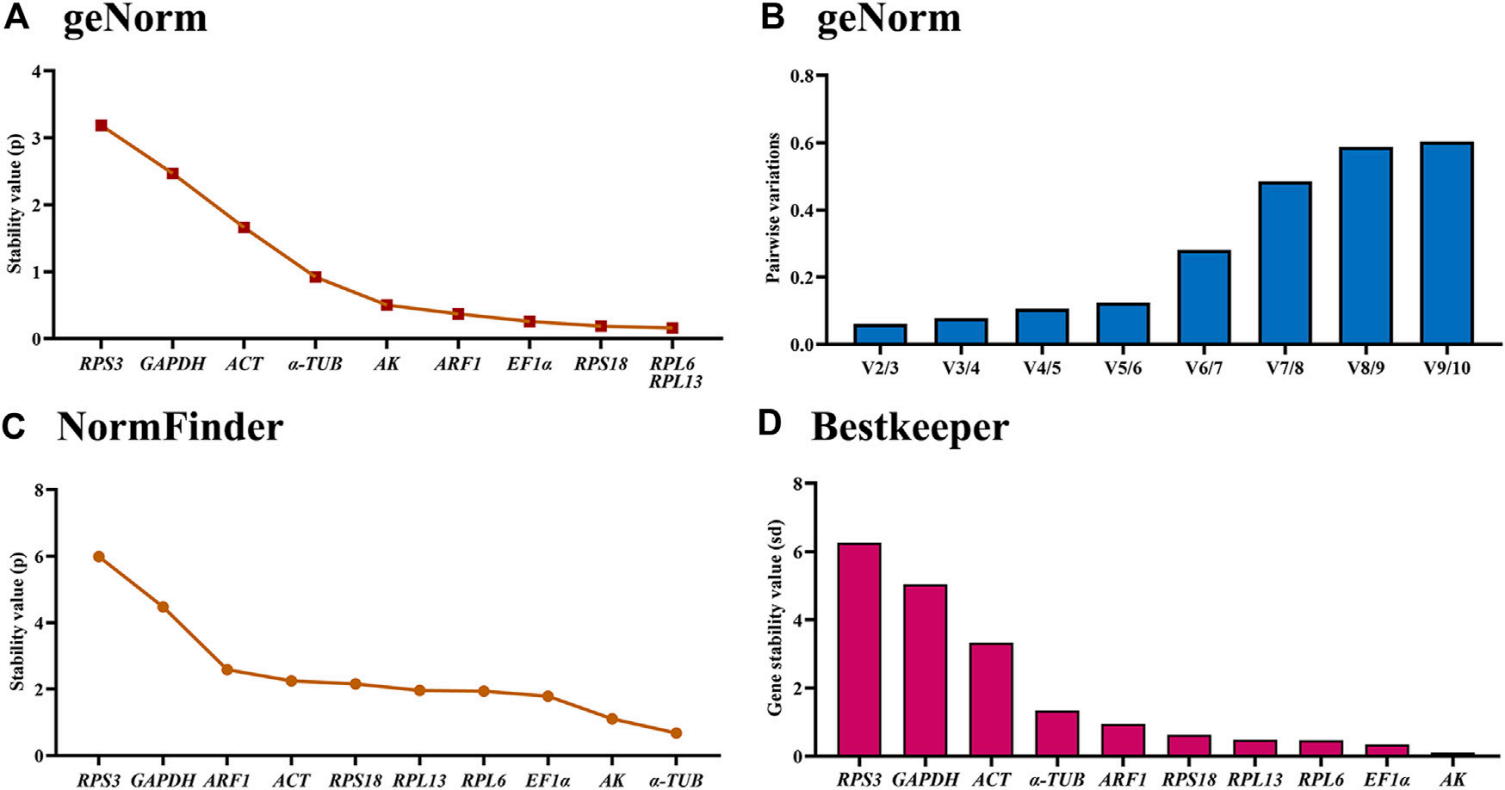
Stabilities of the ten house-keeping genes in *Mylabris sibirica* in males and females. The male and female adults were collected at day 5 after emerging. The expression stability rankings are determined by geNorm, NormFinder and BestKeeper.

Based on the geNorm algorithm results, *RPL6*, *RPL13*, *RPS18*, *EF1α* and *ARF1* were the most stable, with the M-values less than 0.5 (Figure 5A; Table 2). In addition, pairwise variation data manifested that the V2/3 to V5/6 values were less than 0.15, indicating that two different internal genes are suitable for evaluating gene expression levels in males and females (Figure 5B).

Based on the NormFinder data, the stability rankings were as follows: *α-TUB*>*AK*>*EF1α*>*RPL6*>*RPL13*>*RPS18*>*ACT*>*ARF1*> *GAPDH*>*RPS3* (Figure 5C; Table 2). Again, except for *α-TUB*, the *p* values of other reference genes were above 1.0 (Figure 5C; Table 2).

The BestKeeper result demonstrated that *AK*, *EF1α*, *RPL6* and *RPL13* were the most optimal genes, whose SD values were 0.109, 0.345, 0.469 and 0.484, respectively (Figure 4D; Table 2). Again, values of these genes were less than 0.5, showing their similar stabilities.

**FIGURE 6:**
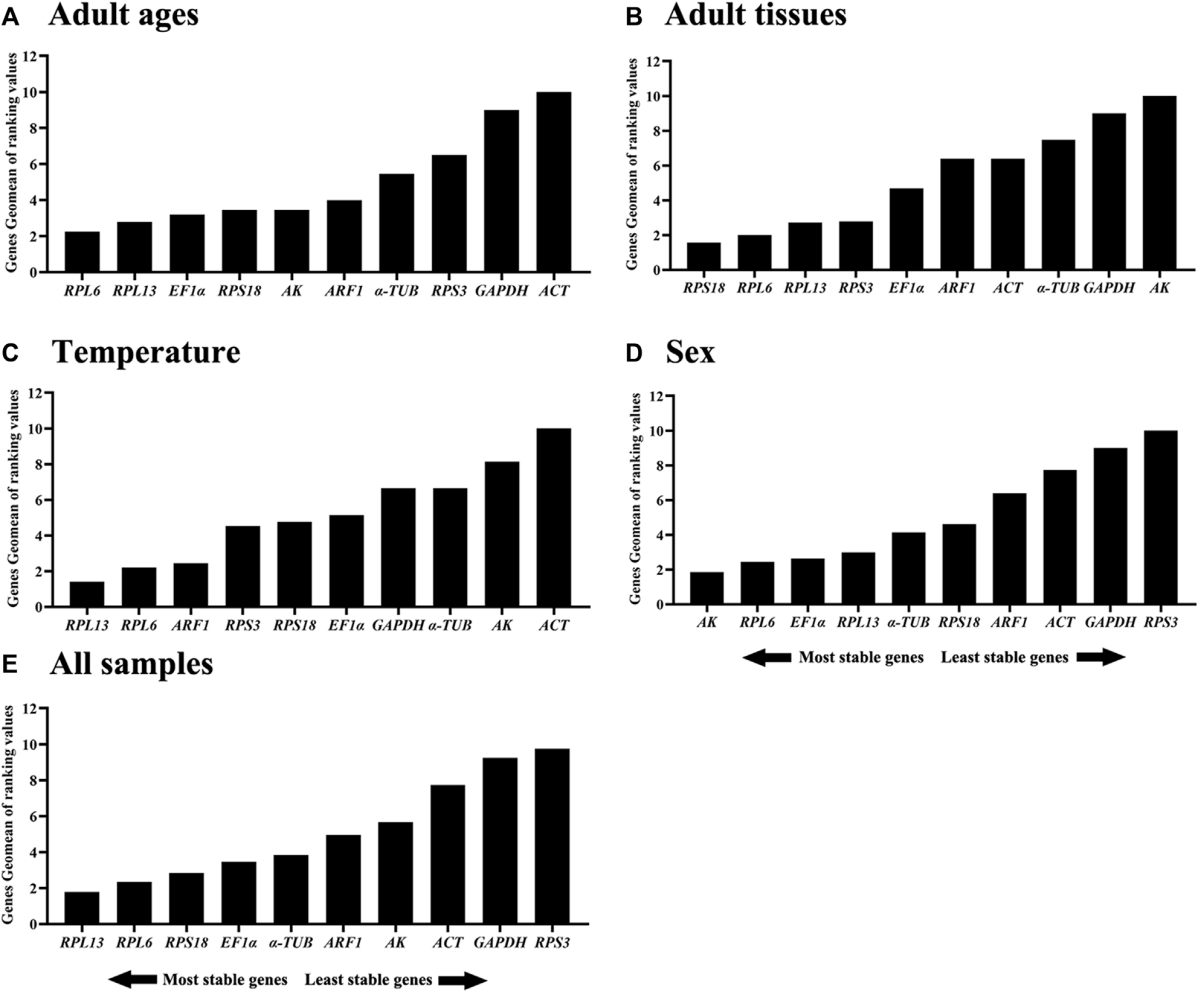
Stabilities of the ten house-keeping genes in *Mylabris sibirica* in different samples. The stability of the reference genes as calculated by the Geomean method of RefFinder. A lower Geomean of ranking value denotes more stable expression. **(A)** Adult ages, **(B)** adult tissues, **(C)** temperature, **(D)** sex, **(E)** all samples.

The RefFinder showed a comprehensive stability ranking: *AK*>*RPL6*>*EF1α*>*RPL13*>*α-TUB*>*RPS18*>*ARF1*>*ACT*>*GAPDH*>*RPS3* (Figure 6D). Therefore, *AK* and *RPL6* are considered as the most optimal internal gene pair (Table 3).

When combining the four diverse experimental conditions together, the RefFinder data showed a overall stability ranking: *RPL13*>*RPL6*>*RPS18*>*EF1α*>*α-TUB*>*ARF1*>*AK*>*ACT*>*GAPDH*>*RPS3* (Figure 6E). In summary, *RPL6* and *RPL13* can be best internal genes for gene expression and microbial abundance determination in *M. sibirica* (Table 3).

### Validation of the selected reference genes

**FIGURE 7:**
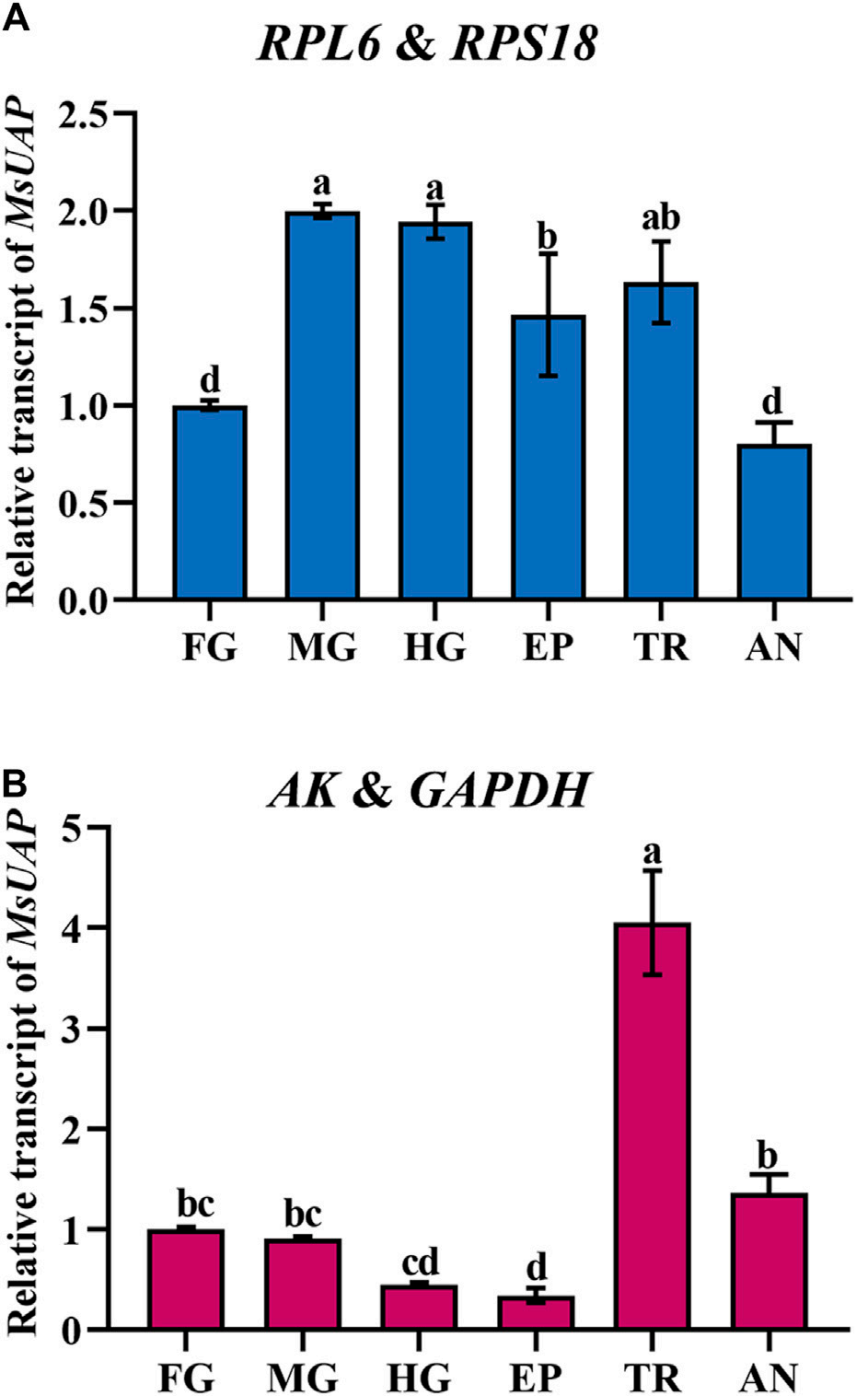
Relative gene expression of *UAP* in different adult tissues of *Mylabris sibirica*. The relative gene expression level of *UAP* in the foregut, midgut, hindgut, epidermis, trachea and antenna was normalized to the best stable [**(A)** *RPL6* and *RPS18*] and least stable [**(B)** *AK* and *GAPDH*] reference genes, respectively. The values are means +SE. Different letters indicate significant differences in gene expression among different tissues (*p* < 0.05).

To assess the stability of the candidate reference genes, the relative expression level of *UAP* was assessed in the epidermis, foregut, midgut, hindgut, trachea and antenna. The following reference genes were used to normalize: *RPL6* + *RPS18* (the most stable reference gene), and *AK* and *GAPDH* (the least stable reference gene). The highest accumulated mRNA level of *MsUAP* was detected in the midgut, hindgut and trachea, followed by those in the epidermis, the lowest level was found in the foregut and antenna. However, *AK* and *GAPDH* was used as reference genes, and *MsUAP* was highly expressed in the trachea, lowly expressed in the epidermis (Figure 7).

## Discussion

Quantitative real-time PCR (qRT-PCR) is a popular method for assessing gene expression (Derveaux et al., 2010; Jozefczuk and Adjaye, 2011) and microbial abundance determination (Ali et al., 2018a; Ali et al., 2018b; Ali et al., 2018c; Ali et al., 2019). Optimal reference genes are critical greatly in eliminating heterogeneity in diverse datasets and improving the quantitative results (Bustin et al., 2009). An ideal internal gene are abundantly expressed under diverse experimental treatments (Derveaux et al., 2010). However, not all internal genes remains suitable in various species (Zhang et al., 2022). Therefore, selecting suitable references must be conducted before qRT-PCR. Studies on internal genes have been reported in many insect species (Shakeel et al., 2018), such as *Aphidoletes aphidimyza* (Shen et al., 2023), *Nilaparvata lugens* (Zhang et al., 2022), *Phthorimaea operculella* (Shen et al., 2022), *H. vigintioctomaculata* (Zhang et al., 2022) and *P. striolata* (Guo et al., 2023). As an oilseed rape pest, evaluating optimal reference genes are helpful in studying molecular mechanisms in *M. sibirica*. As we all knows, the current research is the first report on reference genes assessment in *M. sibirica*.

In the current text, the stability of ten HKGs for *M. sibirica* (*ACT*, *ARF1*, *AK*, *EF1α*, *GAPDH*, *α-TUB*, *RPL6*, *RPL13*, *RPS3* and *RPS18*) were investigated under four experiment treaments (adult ages, adult tissues, temperature and sex), with five methods (Ct value, geNorm, NormFinder, BestKeeper and RefFinder).

Our findings indicated that the best reference gene pairs were *RPL6* and *RPL13* during different adult ages (Figures 1, 2, 6; Table 2) and under diverse temperatures (Figures 1, 4, 6; Table 2), *RPL6* and *RPS18* in various adult tissues (Figures 1, 3, 6; Table 2). Moreover, *RPL6* and *RPL13* were the most stable reference gene pair in tested backgrounds (Figure 6). These results also demonstrated that ribosomal protein genes were the most optimal internal genes to meassur target gene expression in *M. sibirica*. As we all know, ribosomal proteins play an important part in ribosome assembly. They combine with four ribosomal RNAs (rRNAs) to form the ribosomal subunits, which have an important function cellular protein translation (Landry-Voyer et al., 2023). Consistent with our findings, reference genes involving ribosomal protein genes have been selected frequently for expression analysis in various insects during the last 10 years (Zhang et al., 2022; Shen et al., 2022). They are selected as reference genes in Coleopterans *Ips sexdentatus* (*RPS3*) (Sellamuthu et al., 2021), *Tribolium castaneum* (*RPS6*, *RPL13a*, *RPS3* and *RPL18*) (Toutges et al., 2010), *L. decemlineata* (*RP18* and *RP4*) (Shi et al., 2013), *H. vigintioctopunctata* (*RPL13* and *RPS18*) (Lü et al., 2018), *H. vigintioctomaculata* (*RPS18* and *RPL13*) (Zhang et al., 2022) and *P. brassicae* (*RPL32* and *RPL19*) (Ma et al., 2021), Lepidopteran species *Helicoverpa armigera* (*RPS15* and *RPL27*) (Zhang et al., 2015), *Plutella xylostella* (*RPS13* and *RPS23*) (Fu et al., 2013) and *P. operculella* (*RPL13*) (Shen et al., 2022), Thysanopterans *Frankliniella occidentalis* (*RPL32*) (Zheng et al., 2014), Dipterans *Chlorops oryzae* (*RPS15*) (Tian et al., 2019) and *A. aphidimyza* (*RPL8* and *RPS3*) (Shen et al., 2023), Hymenopterans such as *Apis mellifera* (*RPS5* and *RPS18*) (Jeon et al., 2020) and *Anastatus japonicus* Ashmead (*RPL13* and *RPS6*) (Liu et al., 2022), Hemipterans *N. lugens* (*RPL5*, *RPS8* and *RPL14*) (Zhang et al., 2022), *Aphis glycines* (*RPS9*) (Bansal et al., 2012), *Dichelops melacanthus* (*RPL9* and *RPS23*) (Pinheiro et al., 2020), *Diaphorina citri* (*RPL7*) (Bassan et al., 2017) and *Rhopalosiphum padi* (*RPL13*, *RPS6* and *RPS18*) (Li et al., 2021), and Orthopteran *Locusta migratoria* (*RPL32*) (Yang et al., 2014), as well as Acari *Brevipalpus yothersi* (*RPL13* and *RPL32*) (Rogerio et al., 2019).

In male and female adults, the most reliable reference genes were *AK* and *RPL6* (Figures 1, 5, 6; Table 2). As we discussed in the previous paragraph, *RPL6* was expressed abundantly in males and females. Arginine kinase (AK) has a prominent function in invertebrate energy metabolism, which catalyzes the reversible phosphorylation of l-arginine to make up phosphoarginine (Shi et al., 2012). Similar to our results, the arginine kinase gene has been selected as the most suitable gene for expression assessment in *Xylosandrus germanus* (Patwa et al., 2021), *Spodoptera frugiperda* (Han et al., 2021) and *Spodoptera litura* (Lu et al., 2013).

Moreover, The BestKeeper results demonstrated the SD values of *ACT, α-TUB* and *GAPDH* were above 1.0 (Table 2), suggesting that the three reference genes were unsuitable as internal genes for RT-qPCR.

Actin (ACT) is extremely abundant in eukaryotes, and has an vital function in cellular activities, including cell motility and the regulation of transcription cell secretion (Dominguez and Holmes, 2011). At present, many publictions have verified that the expression level of *ACT* is less steady in various insects, such as *P. operculella* (*RPL13*) (Shen et al., 2022), *H. vigintioctopunctata* (*RPL13* and *RPS18*) (Lü et al., 2018) and *Hippodamia convergens* (Yang et al., 2016).

Glyceraldehyde-3-phosphate dehydrogenase (GAPDH) is a key enzyme participating in glycolysis and glucose metabolism (Zhao et al., 2022). Similar to our results, the instability of *GAPDH* expression has reported in *Ophraella communa* (Zhang et al., 2020) *P. brassicae* (Ma et al., 2021) and *Colaphellus bowringi* (Tan et al., 2015).

Microtubule α-tubulin (α-TUB), interacts with many microtubule-associated proteins to conduct a variety of cellular functions, such as intracellular transport and cell division (Hammond et al., 2008). Currently, many studies indicated that the stability of *GAPDH* expression was greatly unsteady in *P. operculella* (Shen et al., 2022), *H. vigintioctomaculata* (Zhang et al., 2022), *N. lugens* (*RPL5*, *RPS8* and *RPL14*) (Zhang et al., 2022) and *A. aphidimyza* (Shen et al., 2023), In brief, our results recommend *RPL6* and *RPL13* as the most stable internal gene pair under four tested backgrounds (Figure 6; Table 3). To further validate the reference genes in *M. sibirica*, we assessed the relative expression level of *UAP* in different adult tissues. Our findings revealed that *UAP* expression pattern was inconsistent in the different adult tissues when normalized to the two best- and least-stable reference genes (Figure 7). These results showed that the unreasonable use of reference genes may lead to inaccurate results for target genes. Therefore, it is crucial to choose and validate the best reference genes to ensure the accuracy of gene expression. Our study would provide a foundation for further gene molecular functions and microbial abundance determination in *M. sibirica*.

## Data Availability

The datasets presented in this study can be found in online repositories. The names of the repository/repositories and accession number(s) can be found in the article/Supplementary Material.
