# Supplementary material for: Evaluation of reference genes for quantitative expression analysis in Mylabris sibirica (Coleoptera, Meloidae)
Source: Front Physiol. 2024 Apr 8;15:1345836. doi: 10.3389/fphys.2024.1345836 (PMC11033477; doi:10.3389/fphys.2024.1345836)
Supplement: Supplementary file 1 [file Table1.docx]

**Table S1. A list of primers used for RT-PCR of the genes**

| **Gene name** | **Primer sequences (5'to 3´)** | **Amplicon size (bp)** | **Accession number** |
| --- | --- | --- | --- |
| ***ACT*** | Forward TGTAAGGCTGGTTTTGCT  Reverse ACATTTGTTGGAAGGTTGA | 1018 | OR670239 |
| ***ARF1*** | Forward TATTGGGCTTAGATGGTGC  Reverse TTCCATTGCCATATCTAAAC | 449 | OR670247 |
| ***AK*** | Forward TTCGGAGAAATCCCAACA  Reverse TGGTTGGACAGAAAGTTAGG | 1256 | OR670249 |
| ***α-TUB*** | Forward AATGCCATCTGATAAGACTGT  Reverse GGAATCCATGCCAACTTCT | 1213 | OR670241 |
| ***GAPDH*** | Forward CCGTATTGGTCGTTTAGTATT  Reverse TGTTCATCTGTATAACCCAAA | 801 | OR670240 |
| ***EF1α*** | Forward ACAACCACTGGGCATTTA  Reverse CAGCCTTTGTGACTTTTCC | 1300 | OR670242 |
| ***RPL6*** | Forward AAAACTCCAGGAAAATCTAATG  Reverse CCAAACATTGCTGATAGATAA | 752 | OR670248 |
| ***RPL13*** | Forward GCCCCAAAAGGGAATAAT  Reverse GCTTTTACAGCTTTTGGTG | 653 | OR670246 |
| ***RPS3*** | Forward GCAAGTTCGTCGGTGATG  Reverse AACGACTTCCTCTTTTGGT | 629 | OR670243 |
| ***RPS18*** | Forward ACATTCTTCGTATTTTGGG  Reverse GCCTGTTGTTTTGGTATGT | 389 | OR670244 |

Note: ACT, actin; ARF1, ADP ribosylation factor 1; AK, arginine kinase; α-TUB, α-tubulin; GAPDH, glyceraldehyde-3-phosphate dehydrogenase; EF1α, elongation factor 1α; RPL6, RPL13, RPS3 and RPS18, ribosomal protein.
